# Professor Syme, of Edinburgh

**Published:** 1848-09

**Authors:** 


### THE MEDICAL EXAMINER.

PHILADELPHIA, SEPTEMBER, 1848.

### PROFESSOR SYME, OF EDINBURGH.

It will be recollected that, on the decease of Mr. Liston, Mr. Syme of the University of Edinburgh, was elected to his place in University College and University College Hospital, London. This, although regarded as an excellent appointment, gave great offence to the London Surgeons, and especially the Alumni of the College, who believed that there were among them equally competent individuals whose claims had been overlooked. Under these circumstances, and to avoid being a party to the angry controversies going on among the officers of the College and the profession, Professor Syme, although he had removed his residence to London, immediately resigned and returned to Edinburgh, where he has resumed his duties as Surgeon of the Infirmary, and is a candidate for re-election to the chair of Surgery in the University of that city. The step which the distinguished Scotchman has taken is honourable to himself, and will be eminently advantageous to the institutions of which he has long been so great an ornament. No appointment has yet been made to the vacant chair in London, and as it will now probably be confined to the Surgeons of that city, who appear to be about as harmonious as the Kilkenny cats, we shall see how much better they will be satisfied. We apprehend they have found it much easier to unite *against a foreigner*, than it will be to unite *in favour of any one of themselves*, and judging from the past, we may expect to see the announcement of the election of the new professor assailed as violently, and, with the exception of the successful candidate and his friends, as unanimously, as in the case of Mr. Syme.

